# Gene expression plasticity facilitates acclimatization of a long-lived Caribbean coral across divergent reef environments

**DOI:** 10.1038/s41598-024-57319-0

**Published:** 2024-04-03

**Authors:** Karl D. Castillo, Colleen B. Bove, Annabel M. Hughes, Maya E. Powell, Justin B. Ries, Sarah W. Davies

**Affiliations:** 1 Department of Earth, Marine and Environmental Sciences, University of North Carolina at Chapel Hill, Chapel Hill, NC USA; 2 Environment, Ecology and Energy Program, University of North Carolina at Chapel Hill, Chapel Hill, NC USA; 3 Department of Biology, Boston University, Boston, MA USA; 4 Department of Marine and Environmental Sciences, Marine Sciences Center, Northeastern University, Nahant, MA USA

**Keywords:** Acclimatization, Caribbean coral, Reciprocal transplant, Transcriptome plasticity, Environmental variability, Environmental specialization, Ecophysiology, Climate-change ecology, Coral reefs

## Abstract

Local adaptation can increase fitness under stable environmental conditions. However, in rapidly changing environments, compensatory mechanisms enabled through plasticity may better promote fitness. Climate change is causing devastating impacts on coral reefs globally and understanding the potential for adaptive and plastic responses is critical for reef management. We conducted a four-year, three-way reciprocal transplant of the Caribbean coral *Siderastrea siderea* across forereef, backreef, and nearshore populations in Belize to investigate the potential for environmental specialization versus plasticity in this species. Corals maintained high survival within forereef and backreef environments, but transplantation to nearshore environments resulted in high mortality, suggesting that nearshore environments present strong environmental selection. Only forereef-sourced corals demonstrated evidence of environmental specialization, exhibiting the highest growth in the forereef. Gene expression profiling 3.5 years post-transplantation revealed that transplanted coral hosts exhibited profiles more similar to other corals in the same reef environment, regardless of their source location, suggesting that transcriptome plasticity facilitates acclimatization to environmental change in *S. siderea.* In contrast, algal symbiont (*Cladocopium goreaui*) gene expression showcased functional variation between source locations that was maintained post-transplantation. Our findings suggest limited acclimatory capacity of some *S. siderea* populations under strong environmental selection and highlight the potential limits of coral physiological plasticity in reef restoration.

## Introduction

Ecosystems across the globe are facing unprecedented habitat loss, population declines, and changes to behavior and phenology due to anthropogenic climate and ocean change^1,2^. As a result, marginal environments that experience conditions akin to those projected under global change are garnering attention as potential sources of environmentally tolerant populations that may facilitate evolutionary rescue^3^. Organisms may thrive in marginal environments due to phenotypic plasticity, adaptive genetic variation, or their interaction. Whether plasticity or adaptation is favored can depend largely on the scale of environmental variability, the relative fitness of plastic (generalist) and adaptive (specialist) genotypes, the relative proportions of plastic and adaptive genotypes within a population, as well as patterns of population structure and dispersal rates^4^. Environmentally tolerant individuals have the potential to rescue populations experiencing declines if they can maintain plasticity across environments or have wide enough adaptive niches to allow for survival in novel environments.

Our understanding of how adaptive divergence and phenotypic plasticity interact to influence organismal responses to climate change across spatial gradients–particularly for marine ecosystems–remains elusive. Emerging data suggest that local adaptation (*i.e.*, higher fitness in home environments relative to genotypes originating from other environments^5^) may be more prevalent in marine species than previously appreciated^6^. However, acclimatization (*i.e.*, phenotypic response to multiple stressors simultaneously^7^) capacity of marine organisms living in marginal environments, where plasticity is more likely to be favored, can act as an avenue of population persistence as these genotypes may be better primed for projected global change. But plasticity can be energetically costly, so organisms living in more stable environments might rely on more fixed phenotypes^8^. Uncovering the acclimatory, adaptive, and plastic potential of populations across spatial scales will improve our ability to predict the impacts of global change on biological systems and facilitate science-driven ecosystem management to help conserve key species^9,10^.

Reef-building corals are amongst the most globally important marine organisms to human societies due to their role as ecosystem engineers of reef systems, which protect shorelines and support valuable tourism industries. However, coral reef ecosystems are increasingly threatened by global change^11,12^. Thus, unraveling the impacts of changing oceans on coral survival has become a paramount research priority for marine scientists^13,14^. Coral responses to changing oceans vary widely^15–18^, and some populations may possess different capacities to resist and recover after exposure to anomalously warm ocean temperatures^19^. For example, corals originating from more thermally variable inshore sites, which are largely considered to be extreme habitats, exhibit increased thermal tolerance^20–22^ and higher gene expression plasticity^23^ than corals from less thermally variable sites, highlighting phenotypic plasticity as a mechanism for coping with changing environments^23,24^. Further, coral populations can exhibit local adaptation and environmental specialization to their home reef environments^25,26^, although this specialization is likely to incur costs, including limitations to plasticity when conditions change^27,28^. In reality, populations of corals have different capacities for plasticity and adaptation under different scenarios, with these processes depending on the balance between selection and other demographic processes (*e.g.,* migration, population size, etc.). Regardless, a more comprehensive understanding of the adaptive and acclimatory potential of coral populations across reef environments will improve predictions of coral reef resilience to continued global change.

Here, we conducted a four-year three-way reciprocal transplant experiment of the massive Caribbean coral *S. siderea* across three distinct reef environments in southern Belize (nearshore reef, backreef, forereef) to investigate the adaptive and plastic potential of these coral populations. Given that nearshore coral populations are exposed to the greatest environmental variability (*e.g.*, fluctuations in seawater temperature, turbidity, nutrient concentration, and light), we hypothesized that these highly variable habitats (i.e., marginal) select for corals with increased plasticity, which would lead to corals originating from these reefs exhibiting greater health across a variety of reef environments. Similarly, backreef coral populations experience moderate environmental variability that likely generates intermediate health across reef environments. Lastly, we hypothesized that corals originating from forereef coral populations will exhibit greater local adaptation (*i.e.,* less plasticity) given that these populations are exposed to the most stable environmental conditions where costs to plasticity are higher, which would result in reduced health in more variable reef environments (*i.e.,* nearshore, backreef). To test this hypothesis, we quantified survivorship and calcification rates of reciprocally transplanted corals. Calcification rate is an important phenotype of tropical reef ecosystems because of its role in constructing the three-dimensional framework of that ecosystem^29^. These data were complemented with whole-genome gene expression profiling of the coral host and algal symbiont (*Cladocopium goreaui*) to detect molecular signatures and gene expression plasticity metrics associated with phenotypic responses three and a half years post-transplantation.

## Materials and methods

### Site description and temperatures across reef environments

**Figure 1:**
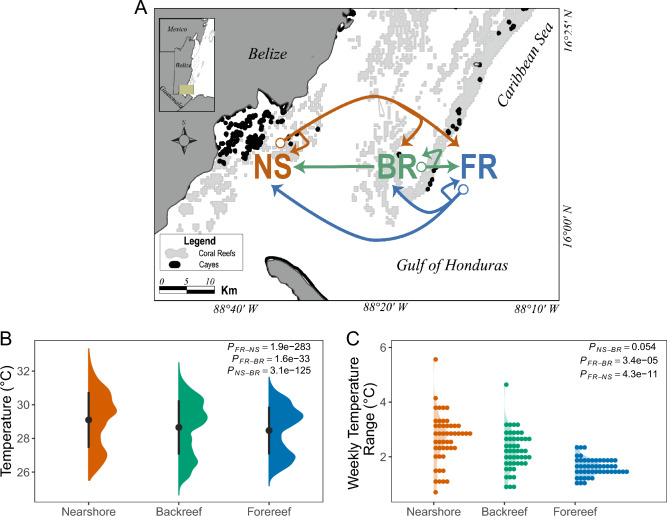
Map of reef environments on the southern Belize Mesoamerican Barrier Reef System with in situ seawater temperatures recorded in these locations. (**A**) Map showing locations of forereef (FR; blue), backreef (BR; green), and nearshore (NS; orange) reef environments, with arrows showing directions of coral transplantation. Forereef and backreef environments are ~ 2 km apart on the seaward and landward sides of the barrier reef’s crest, respectively. The NS site is located 30-km west toward mainland Belize. (**B**) Mean (± 1 SD) and distribution of in situ seawater temperatures taken every 30 min at FR (blue), BR (green), and NS (orange) environments from November 2014 to October 2015. (**C**) Weekly temperature ranges across the three reef environments demonstrating differences in temperature variability across sites.

This research was conducted on the southern terminus of the Belize Mesoamerican Barrier Reef System (MBRS) between a nearshore (NS; 16°11′22.3′′ N 88°34′21.6′′ W) reef located within the Port Honduras Marine Reserve (PHMR), and two more offshore reefs (backreef [BR], 16°07′31.4′′ N 88°16′06.1′′ W, forereef [FR], 16°07′02.9′′ N 88°15′26.2′′ W) located within the Sapodilla Cayes Marine Reserve (SCMR) (Fig. 1A). These three sites were selected because of their unique environmental conditions (*i.e.,* temperature) that were previously characterized from a combination of in situ instrumental and satellite data^30,31^. We supplemented these former observations with high resolution in situ instrumental seawater temperature (Fig. 1B, C; Supplemental Fig. S1) measurements within these environments using Hobo Water Temperature Pro V2 or Hobo Pendant Temperature data loggers (*Onset Computer Corporation;* Bourne, Massachusetts). Loggers were installed in November 2014 (year 3) at 3–5 m depth and were programmed to record temperature at 30 min intervals for approximately one year (until October 2015; year 4). Mean temperatures across the entire sampling period, per month, per week, and per day were calculated at all sites. Further, the monthly, weekly, and daily temperature ranges were calculated to assess the temperature variability at each site. These temperature metrics for each site were compared using Kruskal–Wallis tests and Dunn's Test for Multiple Comparisons with a Bonferroni *p*-value correction.

### Reciprocal transplant experiment

In June 2011 (year 0), 18 colonies (20–30-cm-diameter) of *S. siderea* were collected at a depth of 3–5 m from within each nearshore (NS), backreef (BR), and forereef (FR) environment (Fig. 1A). Each colony was collected from areas > 10 m apart to randomize micro-environmental effects, attain more representative responses within each reef environment, and reduce the probability of sampling clones. In total, 54 *S. siderea* colonies were collected (18 colonies × 3 reef environments).

Entire colonies were removed from the substrate using a hammer and chisel and were affixed to 20 cm × 20 cm commercial tiles pre-labeled with numbered plastic tags using A-788 Splash Zone Marine Epoxy (*Z-Spar*). Colonies were returned to their source reef for recovery for approximately 15 days before the start of the reciprocal transplant experiment. Then all colonies were cleaned of epibionts, photographed, and initial buoyant weight measurements were conducted using a portable buoyant weighing system. Each coral specimen was suspended by an aluminum wire from a *Cole-Parmer* bottom-loading scale (precision ± 0.001; accuracy ± 0.002) at 25 cm depth in a large acrylic aquarium filled with seawater maintained at 28 °C using an aquarium heater. Salinity was maintained at 35 ppt. A glass mass standard was intermittently weighed to ensure consistency of mass measurements across colonies, reef environments, and time.

Colonies were randomly selected and reciprocally transplanted amongst the three reef environments at a depth of 3–5 m. Six NS-sourced colonies were transplanted back to the NS, six were transplanted to the BR, and six were transplanted to the FR. This transplantation procedure was replicated for BR-sourced and FR-sourced colonies for a full three-way reciprocal transplant experiment. Colonies were buoyantly weighed following Davies^32^ annually for four consecutive years in June 2012 (year 1), 2013 (year 2), 2014 (year 3), and 2015 (year 4). Coral survival rates were quantified annually across the four-year experiment and colonies were classified as “alive” if any living tissue was found and were classified as “dead” if no living tissue remained.

The buoyant-to-dry weight relationship for *S. siderea* corals from all environments was empirically derived by plotting dry weights (after removal of organic matter) against buoyant weights of 60 *S. siderea* specimens randomly selected from the same reef environments from a previously published study (R^2^ = 0.9985)^33^. Specimens from all environments were highly correlated, and coral skeleton density did not vary appreciably amongst reefs. Thus, a single linear equation was used to convert buoyant weight to dry weight to estimate net *S. siderea* calcification rates for each of the three reef environments:

$$Dry\, weight \left( {mg} \right) = 1.5567 \times Buoyant\, weight \left( {mg} \right) + 1.1235$$

The resulting dry weights were used as a proxy for net calcification rates of the *S. siderea* colonies from each reef environment by estimating the annual change in each coral’s dry weight normalized to its surface area. The surface area of each coral specimen was quantified using the aluminum foil technique once at the end of the experimental period (year 4)^34^*.*

### Assessment of local adaptation and environmental specialization

To assess local adaptation, we followed Kawecki and Ebert’s^5^ framework that considers the performance of genotypes in their source or transplant environments. Specifically, this framework expects that genotypes will perform better in their source (home) environment than those originating from another (away) environment. Additionally, both populations should possess greater fitness in their respective source environments (local) than in transplant locations (foreign). However, due to our low sample size and use of full coral colonies that confounds genetic variation, we are unable to meet the strictest interpretation of these criteria for local adaptation. Given this, we use the term ‘environmental specialization’ to describe situations where our data suggest higher coral performance in local versus foreign and source versus transplant location situations. Therefore, environmental specialization can be based on the assessment of a single trait (*i.e.*, calcification rates or survival) or a combination of traits, but not all traits need to follow the pattern to showcase evidence of environmental specialization.

### Statistical analyses of survival and calcification rates

Survival of corals throughout the experiment was assessed using a Kaplan–Meier model to visualize survival and an additive Cox proportional hazard model to assess risk significance. Survival data were visualized using the survival function *survfit* from the *survival* package (v2.39-5)^35^.

Calcification rates were estimated using the best-fit, fully interactive linear model to determine the effect of source location, transplant location, and year on calcification rates (Supplementary Table S1). Parametric bootstraps were performed using 4000 iterations to model 95% confidence intervals, with non-overlapping confidence intervals interpreted to represent statistically significant differences in calcification rates. Data were then visualized using package *ggplot2* (v3.2.1)^36^. All statistical analyses and data visualizations for survival and calcification rates were implemented using R software v 3.6.3^37^, with accompanying data and code available on GitHub (https://github.com/seabove7/BelizeRT_Castillo_Bove)^38^.

### Gene expression profiling after 3.5 years of transplantation

In November 2014 (year 3; 3.5 years post-transplantation), colonies were brought to the surface and small tissue microsamples were immediately collected from all living transplant specimens (N = 44). Microsamples from all three sites were collected between 9 and 11 am CDT to control for diurnal variations in expression^39^. Microsamples were preserved immediately in RNAlater, maintained on ice, and then frozen at − 20 °C until RNA was isolated.

Total RNA from 44 individuals (all surviving transplanted colonies at 3.5 years) were isolated using RNAqueous kits (*Ambion*, Life Technologies) and samples were DNAse treated. Approximately 1 µg of RNA *per* sample was prepared for tag-based RNA-seq following Meyer et al.^40^, with several modifications to account for the transition to the Illumina sequencing platform^26,41^. A total of 44 libraries were successfully prepared and sequenced on the Illumina HiSeq version 2500 at UT Austin’s Genome Sequencing and Analysis Facility (GSAF) platform yielding single-end (SE) 50 bp reads (Supplementary Table S2). Raw reads for all samples are available on the NCBI Sequence Read Archive (SRA) under BioProject number PRJNA938378.

A total of 440.8 million raw reads were generated, with individual library counts ranging from 6.5 to 13.3 million reads *per* sample (mean = 10.0 million reads). The fastx_toolkit^42^ was used to remove 5’-Illumina leader sequences and poly(A)^+^ tails. Sequences < 20 bp in length with < 90% of bases having quality cutoff scores < 20 were also trimmed. In addition, PCR duplicates were identified and removed from all libraries. After filtering for quality, 1.8 to 3.7 million reads per sample remained (mean = 2.6 million per sample) and these resulting quality-filtered reads were mapped using Bowtie2.2.0^43^ to a concatenated holobiont reference transcriptome consisting of *S. siderea* and *Cladocopium goreaui* transcriptomes from Davies et al.^24^. This mapping procedure allows for confident parsing of host and symbiont reads and discards reads mapping equally well to contigs in both references or reads that map to neither reference. Only host and symbiont isogroups with a basemean of at least 3 were maintained in downstream analyses. A total number of 21.7 million reads mapped to the *S. siderea* transcriptome with mapped reads per sample ranging from 301,870 to 743,124 (mean 492,969 per sample). For *C. goreaui*, far fewer reads were mapped (11.8% of mapped reads), with a total of 2.9 million reads ranging from 23,375 to 168,102 reads per sample (mean 65,789 reads per sample; Supplementary Table S2). It should be noted that in Tagseq data, each read corresponds to a unique transcript because degenerate primers are incorporated into cDNA synthesis and all PCR duplicates are removed in the data processing steps described above. Therefore even low count data, such as those generated for *C. goreaui* here, can be informative.

### Differential gene expression and Gene Ontology enrichment analysis

All gene expression (GE) analyses were performed in R v. 3.4.2^37^ and analyses for the host and symbiont were conducted in the same way, with the exception that 4 algal symbiont samples (Ssid401, Ssid447, Ssid467, Ssid498) were removed due to low counts (Supplementary Table S2). GE analyses were performed with *DESeq2* v. 1.16.1^44^ and numbers of differentially expressed genes (DEGs) were determined using the model: *design* =  ~ *source* + *transplant*. Counts were normalized and independent pairwise contrasts were computed across all three reef environments for both source and transplant locations separately for hosts and symbionts. Genes identified as differentially expressed were then corrected for multiple testing using the Benjamini and Hochberg false discovery rate (FDR) correction^45^ (adjusted *p*-value < 0.10). Venn diagrams for up- and downregulated genes were generated for all pairwise comparisons and results for all annotated DEGs were visualized using the R package *pheatmap* (v1.0.12)^46^.

To evaluate overall GE patterns between colony source and colony transplant sites, raw host and symbiont data were used as input for independent canonical correspondence analyses (CCAs, alternatively referred to as constrained correspondence analysis), where expression patterns are constrained by source and transplant sites, similar to constrained analyses of gene expression in Armstrong et al.^47^, using the package *vegan* (version 2.5-4)^48^. Raw counts were normalized to total counts and then data were log-transformed using the *decostand* function. Overall significance of constraints (source, transplant) were assessed using PERMANOVAs using the *adonis* function with 999 permutations.

Gene expression plasticity in response to transplantation was calculated following Bove et al.^49^ using the first two CCA axes. Plasticity was calculated as the distance between a coral and the mean of all corals originating from the same source reef that were transplanted back to their original source reef. The effect of transplantation on calculated distances was assessed using generalized linear models (function *glm*) with a Gamma distribution and log-link. The best-fit model was selected as the model with the lowest Akaike information criterion (AIC) (Supplementary Table S3). Parametric bootstraps were performed to model mean response and 95% confidence intervals with 1500 iterations and significant effects were defined as non-overlapping confidence intervals. Marginal and conditional R^2^ values of the best fit models were calculated using the *r2_nakagawa* function in the *rcompanion* package (v 2.4.1)^50^.

Gene ontology (GO) enrichment analyses were performed using Mann–Whitney U tests on ranked *p*-values (GO-MWU^51^) between all pairwise source and transplant locations. GO enrichment for all pairwise comparisons in both host and symbiont were separately tested in the three overarching divisions of ‘cellular component’ (CC), ‘biological process’ (BP), and ‘molecular function’ (MF). Results were plotted as dendrograms with hierarchical clustering of GO categories based on shared genes. Scripts, gene annotation and GO files, raw mapped count data count, and *DESeq2* and GO enrichment results for both the host and the algal symbiont can be accessed as supplemental files at https://github.com/seabove7/BelizeRT_Castillo_Bove.

### Population structure of host and algal symbionts

To determine if population genetic structure or cryptic species (e.g., Rippe et al.^52^) existed across NS, BR, and FR environments within *S. siderea* or *C. goreaui*, sample reads were mapped separately to the *S. siderea* and *C. goreaui* transcriptome references^24^ using Bowtie2 v2.2.0^43^ with default parameters, and then converted to BAM format using SAMTOOLS v1.9. ANGSD v0.935^53^ then genotyped and identified single nucleotide polymorphisms (SNPs). Loci were retained that were present in at least 80% of individuals, had a depth of coverage > 5 reads, a minimum mapping quality score of 20, a minimum quality score of 25, a strand bias p-value > 1 × 10^–5^, a heterozygosity bias > 1 × 10^–5^, a SNP p-value of 1 × 10^–5^, excluded all triallelic sites, removed reads with multiple best hits and passed the lumped paralogs filter. Population structure was explored for the host (4797 SNPs) using three methods: (1) hierarchical clustering of pairwise IBS values; (2) principal component analysis (PCoA) based on the IBS matrix; and (3) admixture v1.3.0^54^ analysis using the optimal K method implemented in Plink v1.90b6.4^55^. Although we explored algal symbiont structure, very few SNPs were detected (405 SNPs) due to low depth of coverage even when the depth of coverage filter was lowered to > 2 reads.

For *S. siderea* host SNP data (N = 4797 SNPs)*,* we first identified 4 outlier samples via PCoA on the IBS matrix (Ssid464, Ssid470, Ssid471, Ssid472). These samples were removed and ANGSD was rerun (N = 4511 SNPs), which then identified one pair of samples that appeared to be strongly related and putative clones (Ssid413, Ssid476). One sample was randomly chosen for removal (Ssid413) and ANGSD was rerun a final time (N = 4655 SNPs) and no outliers were detected. Data were visualized using PCoA on the IBS matrix using *capscale* function from the *vegan *package^48^. Data were further visualized using canonical correspondence analysis (CCA, alternatively referred to as constrained correspondence analysis) where pairwise IBS values were constrained by source site and overall significance of source site was assessed by PERMANOVA using the *adonis2* function with 999 permutations. These same analyses were performed on the algal symbiont data, although we could not estimate an optimal K due to too few sites.

### ITS2 metabarcoding of algal symbiont communities

DNA was extracted using a modified phenol–chloroform protocol^56^ and the ITS2 region of algal symbionts was targeted with the *SYM_VAR_5.8S2 and SYM_VAR_REV* primers^57,58^ using the following PCR profile: 26 cycles of 95 °C for 40 s, 59 ℃ for 2 min, 72 ℃ for 1 min and a final extension of 72 ℃ for 7 min. PCR products were cleaned using the GeneJET PCR Purification kit (*ThermoFisher Scientific*) according to the manufacturer’s instructions. A second PCR was performed using the same profile, but for only 5 cycles to dual-barcode samples. Samples were pooled based on the visualization of band intensity on a 1% agarose gel. The pooled library was then run on a 2% SYBR Green gel, after which the target band was excised sequenced on Illumina MiSeq (paired-end 250 bp) at Tufts Genomics Core Facility.

Illumina adapters and degenerate leaders sequences were trimmed using *bbmap*^59^ and then raw paired-end fastq.gz files were submitted to *Symportal*, which identifies sets of intragenomic ITS2 sequence variants (DIVs) to determine ITS2 type profiles^60^. These data were then visualized in a barplot created using *phyloseq*^61^ to compare relative abundance of majority ITS2 types across corals (Supplementary Fig. S9). Raw reads for all ITS2 samples are available on the NCBI SRA under BioProject number PRJNA938378.

## Results

### Temperature variation across reef environments

In situ temperatures across reef environments from November 2014 to October 2015 (year 4–5) were significantly different (*p* = 3.7 × 10^–29^), with the NS environment experiencing the warmest temperatures (29.1 °C ± 1.66 SD), followed by BR (28.7 °C ± 1.61 SD), and then FR (28.5 °C ± 1.42 SD) (Fig. 1B; Supplemental Fig. S1A–D). NS and BR environments experienced similar temperature ranges at the daily (*p*_*DAY*_ = 0.83), weekly (*p*_*WEEK*_ = 0.054), and monthly temporal scales (*p*_*MONTH*_ = 0.62), while the FR experienced significantly lower temperature variation than the other environments (Fig. 1C; Supplemental Fig. S1E, F). These patterns suggest that FR environments have more stable and cooler seawater than NS and BR environments (Fig. 1B, C; Supplemental Fig. S1). While these in situ measurements only span one year of the reciprocal transplant experiment, previous work monitoring SST in this region over the last several decades via satellite products demonstrates that these temperature patterns are consistent across time for these reefs^62,63^. Additionally, these satellite SST data suggest that these reef locations did not experience thermal anomalies during the experimental period.

### Survival of *Siderastrea siderea* colonies across reef environments

**Figure 2:**
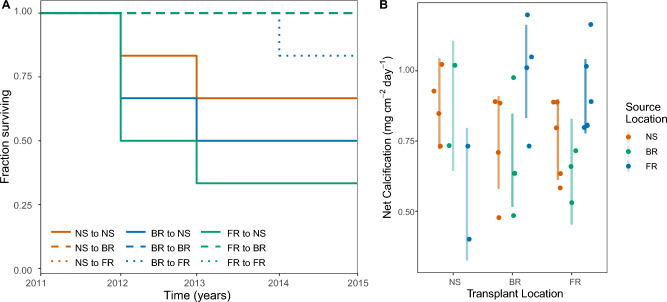
Colony survival and net calcification rates of *S. siderea*. (**A**) Fraction of surviving colonies through time (year 0–year 4) after reciprocal transplantation with respect to source and transplant locations. Transplant location is represented by line type (NS [nearshore] = solid; BR [backreef] = dashed; FR [forereef] = dotted) and source location is denoted by color (NS = orange; BR = green; FR = blue). (**B**) Net calcification rate (mg cm^–2^ day^–1^) averaged over the entire experimental period (year 0–year 4) by transplant location. Source location is represented by color: NS = orange, BR = green, and FR = blue. Colored bars represent modeled 95% confidence intervals with corresponding raw net calcification rates per colony denoted by circles of the same color.

Forty-four of the 54 transplanted colonies (81.5%) survived the four-year (June 2011-June 2015) reciprocal transplant experiment (Supplementary Table S4). Colony source location did not have a significant impact on coral survival throughout the experiment (*p*_SOURCE_ = 0.44). However, transplant location significantly affected coral survivorship (*p*_TRANSPLANT_ < 0.001; Supplementary Table S5A). Specifically, NS-transplanted corals exhibited significantly lower survival than FR-transplanted corals (*p* < 0.05; Supplementary Table S5B). Survival at the FR and BR were statistically indistinguishable from one another, with BR-transplanted corals exhibiting 100% survival regardless of source location (*p* > 0.05; Fig. 2A). Similarly, NS-sourced and FR-sourced corals maintained 100% survival when transplanted to the FR, while a single BR-sourced colony died in year 3 on the FR (83%, 5/6 survivors; Fig. 2A). NS-transplanted corals exhibited the lowest survival regardless of source location. NS-sourced corals exhibited 83% survival in year 1 (5/6 survivors) before dropping to 67% in year 2, after which survival stabilized for the remainder of the experiment. BR-sourced and FR-sourced corals transplanted to the NS also exhibited similar patterns of survival with mortality events in year 1 (BR: 67%, 4/6 survivors; FR: 50%, 3/6 survivors) and year 2 (BR: 50%, 3/6 survivors; FR: 33%, 2/6 survivors) before stabilizing throughout the remainder of the experiment (Fig. 2A).

### Coral calcification rate across reef environments

Differences in *S. siderea* calcification rates were assessed over the last three years of the experiment (year 2–year 4) by transplant location (Fig. 2B; Supplementary Fig. S2C) and source location (Supplementary Fig. S2A, B). Overall calcification rates were not clearly different across NS-sourced or BR-sourced corals when transplanted to any of the three reef locations based on modeled 95% confidence intervals (Fig. 2B; Supplementary Tables S6–S9). Conversely, FR-sourced corals grew slower when transplanted to the NS than when transplanted to the BR (44% slower) or FR (32% slower) (Fig. 2B, Supplementary Table S6). Indeed, FR-sourced corals exhibited a trend towards faster calcification rates compared to the NS- and FR-sourced counterparts, except after transplantation to the NS (Fig. 2B).

### *Siderastrea siderea* gene expression response 3.5 years post-transplantation

**Figure 3:**
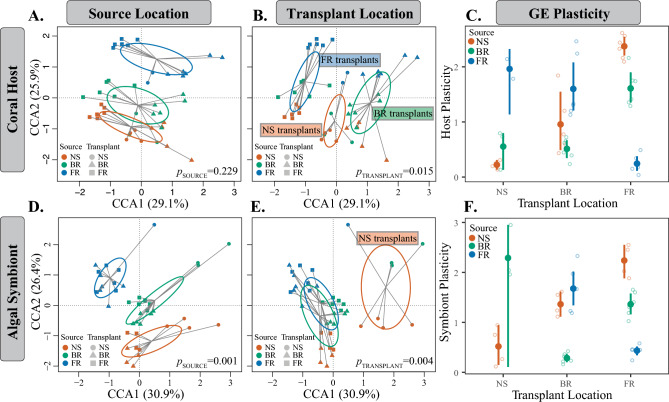
Overall patterns of gene expression for *S. siderea* (**A**–**C**) and *C. goreaui* algal symbionts (**D**–**F**). Canonical correspondence analysis (CCA) of all log-transformed isogroups clustered by (**A**, **D**) source location and (**B**, **E**) transplant location, demonstrating significantly different gene expression patterns for *S. siderea* by transplant (*P*_*TRANSPLANT*_ = 0.015), but not by source location (*P*_*SOURCE*_ = 0.229) and for *C. goreaui* for both source (*P*_*SOURCE*_ = 0.001) and transplant (*P*_*TRANSPLANT*_ = 0.004, nearshore only) locations. (**C**, **F**) Gene expression (GE) plasticity estimates calculated from the first two CCA axes for both hosts (**C**) and algal symbionts (**F**), with transplant location along the x-axis and color representing source location. Calculated GE plasticity is depicted for each sample with open circles, and bootstrapped means and 95% confidence intervals represented by solid circles and vertical bars, respectively.

Overall gene expression patterns of surviving corals 3.5 years after reciprocal transplantation revealed that corals exhibited expression profiles more similar to other colonies transplanted to the same reef environment than to colonies transplanted to different reef environments (*p*_*TRANSPLANT*_ = 0.015; Fig. 3B), with no effect of source location detected (*p*_*SOURCE*_ = 0.229; Fig. 3A). Gene expression plasticity analyses of *S. siderea* determined that FR-sourced corals transplanted to the NS exhibited higher gene expression plasticity than BR-sourced corals transplanted to the NS (Fig. 3C; Supplementary Table S10A-host). FR-sourced corals transplanted to the BR exhibited higher plasticity, while NS-sourced corals had similar plasticity to BR-sourced corals. NS-sourced corals transplanted to the FR exhibited the highest plasticity, with BR-sourced corals also showing high plasticity, although less than NS-sourced corals. However, it should be noted that fewer FR- and BR-sourced colonies survived transplantation to the NS, so this analysis may have lower statistical power than the analysis of NS-sourced specimens.

Numbers of differentially expressed genes (DEGs) for the host exhibited many more DEGS for transplant location relative to source location. The only DEGs detected between source locations were between NS-sourced and FR-sourced corals (5 genes) and no other pairwise comparisons resulted in any DEGs (Supplementary Fig. S3A). There were DEGs detected for all pairwise comparisons across transplant sites with 78 DEGs between NS-transplanted and FR-transplanted corals (20 upregulated in NS, 58 upregulated in FR), 111 DEGs between NS-transplanted and BR-transplanted corals (37 upregulated in NS, 64 upregulated in BR), and 283 DEGs between BR-transplanted and FR-transplanted corals (115 upregulated in BR, 168 upregulated in FR) (Supplementary Fig. S3B). Heatmaps of all annotated *S. siderea* DEGs for transplant location are shown in Supplemental Fig. S4. Of interest, stress and immunity-related genes were upregulated in corals transplanted to the BR relative to NS- and FR-transplanted corals, which included *superoxide dismutase*, *interferon regulatory factor 8,* and several ubiquitin genes. In addition, *TNFAIP3-interacting protein 1* and *NF-kappa-B* p105 were both upregulated in BR-transplanted corals relative to FR-transplanted corals. Heatmaps could not be generated for source location because only one annotated DEG was found (upregulated in NS-sourced corals relative to FR-sourced corals), which was *Multiple PDZ domain protein*.

GO enrichment analyses showed that, regardless of the pairwise comparison, corals living in the FR were enriched for functions associated with ion transport (*e.g.,* ion transport: GO:0006811, anion transport: GO:0006820, transmembrane transport: GO:0055085) and muscle growth (*e.g.,* collagen trimer: GO:0005581, contractile fiber part: GO:0043292, translation: GO:0006412) (Supplementary Fig. S6). In contrast, BR-transplanted corals were enriched for terms associated with stress (*e.g.,* oxidoreductase complex: GO:1990204, nuclear transcription factor complex: GO:0005667) and respiration (*e.g.,* mitochondrial part: GO:0044455, mitochondrial respiratory chain complex I: GO:0005747). Lastly, colonies transplanted to the nearshore were enriched for terms associated with response to stimulus (*e.g.,* response to endogenous stimulus: GO:0009719, cell surface receptor signaling pathway: GO:0007166) and ion transport (*e.g.,* ion transport: GO:0006811, passive transmembrane transporter: GO:0022803) (Supplementary Fig. S5).

### *Cladocopium goreaui* gene expression response 3.5 years post-transplantation

Overall algal symbiont gene expression patterns from surviving *S. siderea* corals revealed contrasting patterns to the host. First, we observed a significant effect of source location even after 3.5 years of transplantation (*p*_*SOURCE*_ = 0.001; Fig. 3D). Algal symbiont gene expression was also significant for transplant location, although this result was driven by algae associated with corals transplanted to the NS (*p*_*TRANSPLANT*_ = 0.015; Fig. 3E). Algal symbiont gene expression plasticity followed a similar pattern to coral host plasticity, although the magnitude of plasticity varied (Fig. 3F; Supplementary Table S10B-symbiont). While this analysis had relatively low statistical power due to low survivorship of NS-transplants and lower depth of coverage, both NS- and FR-sourced algal symbionts associated with corals transplanted to the BR exhibited higher plasticity than BR-sourced algal symbionts also transplanted to the BR. Furthermore, similar to host plasticity analyses, algal symbionts from NS-sourced colonies transplanted to the FR exhibited the highest plasticity.

In contrast to host DEGs, algal symbionts showed more DEGs with respect to source location than transplant location. Even though CCA results suggested that transplant location had a significant effect on overall gene expression, very few DEGs were detected (3 upregulated in FR-transplants relative to BR-transplants) (Supplementary Fig. S3D). There were DEGs detected for all pairwise comparisons across source sites with 29 DEGs between NS-sourced and FR-sourced corals (16 upregulated in NS, 13 upregulated in FR), 9 DEGs between NS-sourced and BR-sourced corals (5 upregulated in NS, 4 upregulated in BR), and 73 DEGs between BR-sourced and FR-sourced corals (28 upregulated in BR, 45 upregulated in FR) (Supplementary Fig. S3C). Heatmaps of all annotated DEGs for source location are shown in Supplemental Fig. S6. Of interest, *C. goreaui* associated with FR-sourced corals exhibited upregulated genes associated with stress (*i.e., Ubiquitin-conjugating enzyme E2, Apoptosis-inducing factor homolog B*) relative to symbionts from BR-coursed corals. We were unable to generate heatmaps for algal data by transplant location because none of the three DEGs for the BR-transplant:FR-transplant comparison were annotated.

GO enrichment analyses showcased that algae from BR-sourced corals consistently exhibited underrepresentation of GO terms associated with photosynthesis. For example, functions associated with the thylakoid (GO:0009579) and photosystem (GO:0009523) were enriched in FR-sourced corals relative to BR-sourced corals (Supplementary Fig. S7). NS-sourced corals also showcased enrichment of those same terms, along with others associated with photosynthesis (*i.e.,* chloroplast part (GO:0019750), tetrapyrrole binding (GO:0046906), chlorophyll binding (GO:0016168)) (Supplementary Fig. S7), relative to BR-sourced corals.

### Host and symbiont genetic structure

For the 39 *S. siderea* host samples that passed quality control (4 identified as outliers, 1 identified as putative clonal ancestry), no genetic structure was detected between sites regardless of the metric of comparison. Admixture identified K = 1 populations, suggesting that corals from these sites belong to the same metapopulation. PCoA analysis confirmed this result (Supplementary Fig. S8A). While CCA results suggested that samples clustered by source site, each axis explained very little variation (CCA1: 2.7%; CCA2: 2.4%) and this clustering was not significant (p = 0.809; Supplementary Fig. S8C). Very few SNPs were identified for the algal symbiont (N = 405) and PCoA and CCA analyses using these data suggest that these *C. goreaui* also belonged to the same metapopulation, although these analyses had relatively low statistical power due to the low number of SNPs detected and the CCA analysis was trending towards significance (p = 0.079; Supplementary Fig. S8B, D).

ITS2 metabarcoding confirmed that all colonies were dominated by *C. goreaui* (ITS2 type C1), with sample 456F hosting background amounts of *Symbiodinium tridacnidorum* (ITS2 type A3) and sample 467 hosting *Breviolum spp.* (ITS2 type B1dk and B5) (Supplementary Fig. S9). Given the low genetic diversity across samples, we were unable to explore diversity beyond the majority ITS2 type. Overall, raw ITS2 counts were 14,750 ± 6574 (mean ± SD) and 4,905 ± 2,435 (mean ± SD) after profiling by *Symportal*.

## Discussion

Theory suggests that predictable temporal environmental variation should favor phenotypic plasticity^64^. We therefore hypothesized that corals from the environmentally variable nearshore (NS) population would exhibit greater transplantation success than those from the more stable and cooler forereef (FR) and backreef (BR) populations^30,31^. Our predictions were also based on previous work on the Belize MBRS in which FR *S. siderea* exhibited increased physiological stress^65^, greater declines in skeletal extension^15^, and reductions in symbiont photophysiology at elevated temperatures^66^ relative to their NS counterparts. Here, we found source location significantly affected calcification rate and symbiont gene expression, but survival and coral host gene expression patterns, which were primarily driven by transplant location. Overall, only FR-sourced *S. siderea* demonstrated evidence for environmental specialization, exhibiting increased mortality when transplanted to the the most marginal site (NS) (Fig. 2A) and higher calcification rates within their home FR environment relative to transplantation to the NS (Fig. 2B). While these data do not fully satisfy the home versus away criteria of local adaptation^5^ since FR-sourced corals did not also incur these fitness costs in BR environments, they do suggest a phenotypic advantage of FR-sourced *S. siderea* populations in their home environment that is lost when transplanted to NS environments.

Our findings are consistent with previous work demonstrating local adaptation in corals from cooler, more thermally stable environments, such as *Porites lobata* in American Samoa^67^ and Palau^68^, and Caribbean *P. asteroids*^20^. Coral populations from these less variable environments may demonstrate higher capacities for environmental specialization because they experience more stable environmental conditions over longer intervals^69^, increasing opportunity for phenotype-environment matching^5,70^. An extreme example of phenotype-environment matching is the presence of cryptic lineages in *S. siderea* along the Florida reef tract, where different cryptic lineages occupy unique niches and exhibit environmental specialization^52^. However, while our coral populations exist across a wide environmental gradient, we found no evidence for cryptic lineages (Supplemental Fig. S8A). However, calling SNPs in short TagSeq data is likely to have limited resolution and deeper whole-genome sequencing in the future may detect genetic divergence between these populations. Regardless, this absence of genetic differentiation suggests that individuals from NS or BR environments are not selected against in the FR environment and they appear to perform equally well across sites, suggesting that NS- and BR-sourced corals exhibit wider plasticity. In contrast, FR-sourced corals appear more specialized to their FR environments on the Belize MBRS and they calcify best in their home environment, consistent with environmental specialization. However, the fact that we find no population differentiation between these populations may also suggest that FR corals are exhibiting long-term acclimatization rather than local adaptation.

Notably, we did not find evidence for environmental specialization in NS- and BR-sourced populations of *S. siderea*. While BR-sourced corals exhibited similar mortality to FR-sourced corals when transplanted to the NS, we did not observe the same growth advantage in their home reef. Further, NS-sourced corals had the lowest survival in their home reef compared to transplant locations and indistinguishable calcification rates across the three reef environments. These patterns are consistent with other reciprocal transplant studies. For example, higher survivorship of *Acropora hyacinthus* transplanted from a highly variable (HV) to moderately variable (MV) habitat was observed relative to those transplanted from MV to HV where greater mortality occurred^71^. However, our results contrast patterns found by Baumann et al.^27^ during a 17-month *S. siderea* reciprocal transplant between more northern reef environments of the Belize MBRS. Here, they found evidence of *S. siderea* local adaptation to more thermally variable inshore environments, and increased capacity for acclimatization by offshore corals to this inshore environment. These differences may be due to experimental design (*e.g.,* duration: 17-months *vs.* four years; transplantation method: fragmented corals *vs.* whole coral transplantation), handling of the corals during experimentation, an anomalously stressful time period in the NS environment, and/or the presence of cryptic lineages^52^ at these more northern locations. The lack of environmental specialization in NS- and BR-sourced coral populations also aligns with the lack of population genetic structure in the SNP data. However, it is worth noting once again that adaptive loci might only be detectable using higher resolution techniques, such as whole genome resequencing (*i.e.*, Fuller et al.^72^) and TagSeq data are likely limited in their power for detecting population structure since genic regions are much more likely to be conserved than other regions of the genome. Future work investigating *S. siderea* population structure using higher resolution approaches is needed to more carefully assess the potential for environmental specialization on the Belize MBRS.

Nearshore reef environments of the Belize MBRS experience warmer temperatures and higher thermal variability (Fig. 1B, C), which is often accompanied by variability of other abiotic factors (*e.g.,* pH, turbidity, oxygen, salinity, sedimentation, light^31,73,74^). These conditions vary in complex ways over short- and long-term cycles and contribute to the relative success of tropical reef-building corals^75^, particularly under changing ocean conditions. Here, we found that NS-transplanted corals exhibited the lowest survival rates, even when sourced from the NS (Fig. 2A). This pattern suggests that the more environmentally challenging NS conditions, such as freshwater input^76^, sedimentation and nutrient loading^77^, as well as variation in temperature^30^, light^78^, and hydrodynamic regimes^79^, negatively impact *S. siderea* survival. Such environmental selection in corals has been observed previously across coral populations^71,80^ and may limit the success of coral restoration efforts in these environments. Of the surviving corals in this experiment, FR-sourced corals transplanted to the NS exhibited reduced calcification (Fig. 2B), which is consistent with previous work transplanting corals to more marginal sites^28,81^. This suggests that while some corals may be able to survive in these NS environments, it may come at a physiological cost^71^. These marginal environments are defined by complex interactions between environmental conditions (*e.g.,* temperature, light, pH, etc.) that significantly influence the growth and success of corals in these locations. For example, although coral calcification rates may be enhanced by nutrient input^82^, eutrophication can also compromise coral growth^82^. Similarly, increased sedimentation can enhance suspension-feeding, especially for corals from high-turbidity environments^83^, but anthropogenic sedimentation can decrease survival^84,85^. It is challenging to attribute reduced coral health in the NS environment to a single environmental parameter, and instead it is more likely that multiple environmental stressors within this environment interact to limit survival and growth in the NS.

We hypothesized that one mechanism corals employ for surviving transplantation across environments is transcriptome plasticity^39^. Here, we observed that *S. siderea* exhibited indistinguishable transcriptome profiles from counterparts at the same reef location 3.5 years after transplantation, regardless of source location (Fig. 3B). These results suggest that if *S. siderea* survive transplantation, they show gene expression plasticity, which facilitates their existence in novel environments. Indeed, a previous 95-day common garden experiment on *S. siderea* from these same sites revealed patterns of gene expression primarily driven by experimental treatment (temperature and *p*CO_2_) and not source environment^24^. Together, these data provide further support for the broad capacity for transcriptome plasticity and might explain, in part, how *S. siderea* are capable of exhibiting such resilience under environmental change^17,86^.

Transcriptome plasticity has been previously observed in coral reciprocal transplant experiments. When colonies of *P. astreoides* were transplanted across environments in Florida, corals exhibited gene expression profiles that shifted to more closely match local corals from the transplant environment over time^23^. However, this capacity for plasticity differed between environments, with inshore-sourced *P. astreoides* being better able to match their forereef counterparts one year post-transplantation. Here, the extent of gene expression plasticity tended to match the scale of environmental difference between source and transplant sites. For example, NS-sourced corals transplanted to the FR exhibited greater gene expression plasticity than BR-sourced corals transplanted to the FR. This pattern likely arises because the BR environment is more similar (with respect to temperature; Fig. 1B, C) to the FR than to the NS, thereby potentially requiring less transcriptome plasticity following transplantation. Although our results suggest that transplanting FR-sourced corals to the NS yields low survivorship, if the FR-sourced coral survives, it exhibits high transcriptome plasticity. It is possible that all FR-sourced corals transplanted to the NS initially exhibited transcriptome plasticity, but this plasticity may have been too costly and led to mortality of certain colonies. However, it is impossible to know the timeframe of this plasticity and the potential costs given our experimental design. Future work should consider sampling gene expression and energetic reserves across multiple time points to better explore the timescale and cost associated with transcriptome plasticity. Alternatively, NS environments could select for genotypes with high plasticity, which would suggest that FR populations have existing standing genetic variation in plasticity that selection can act on. Differences in genetic diversity may also exist across these populations, which may drive these differences in plasticity. Additional work controlling for genotype is needed to disentangle genetic and plastic effects in response to transplantation. Regardless, this capacity for transcriptome plasticity may increase the chance of survival under novel conditions, suggesting that variation in the capacity for plasticity between individuals may be selected for under rapidly changing environments.

One might hypothesize that corals living in NS environments would experience greater stress given that these environments are thermally variable (Fig. 1B, C) and experience more direct anthropogenic impacts (*e.g.*, runoff from land). However, we observed that corals transplanted to the BR, regardless of source location, exhibited an upregulation of genes associated with stress (*e.g., superoxide dismutase*) and immunity (*TNFAIP3-interacting protein 1*, *NF-kappa-B* p105). Regulation of tumor necrosis factors (TNFs; *e.g.*, *TNFAIP3-interacting protein 1*) has been widely associated with coral bleaching and resilience^25,87^. For example, corals exposed to more thermally variable environments maintain constitutively higher TNF expression (*i.e*., gene frontloading), which may facilitate thermal priming to future heat events^25,87^. Higher *NF-kappa-B* expression in corals living on the BR is of particular interest given this transcription factor’s role in cnidarian innate immunity^88^. This pattern suggests that these corals are experiencing immunity challenges and are upregulating this pathway. Further, corals living on the BR exhibited enrichment of GO terms associated with respiration (*e.g.,* mitochondrial respiratory chain complex I). Previous work on *S. siderea* also observed upregulation of respiration pathways associated with maintenance of calcification under ocean acidification conditions^24^. Therefore, our results suggest that corals living on the BR are upregulating genes associated with stress, which may come at a metabolic cost via increased respiration. Alternatively, this upregulation may simply suggest that corals on the BR are experiencing stress. Future assessment of immunity and respiration rates across these sites is warranted to better link genotype to phenotype.

Enrichment for GO terms associated with ion transport and muscle growth were observed in corals transplanted to the FR relative to those transplanted to the BR or NS. Similar GO terms were previously shown to be underrepresented in *S. siderea* under severe heat stress and were correlated with severe reductions in calcification rate^24^. In contrast, *S. siderea* transplanted to the NS exhibited enrichment of GO terms associated with response to stimulus (*e.g.,* cell surface receptor signaling pathway) that may indicate increased heterotrophy in NS environments^89^. While we do not observe greater calcification rates for NS-transplanted corals, it is possible that corals living on the NS have greater tissue biomass and energy reserves, which are important aspects of coral health^90^ that were not quantified here. This trait may be particularly important for corals living in NS environments that experience more variable and turbid conditions, for which higher biomass or energy reserves may facilitate tolerance to such marginal environments^91^.

The type of endosymbiotic algae (family Symbiodiniaceae) with which corals associate can influence coral physiology, growth, thermal tolerance, and even gene expression^92^. Here, we found that *S. siderea* from all environments hosted a majority of *Cladocopium goreaui* (ITS2-type C1; Supplementary Fig. S9). Although no differences in the population structure of algae associated with corals originating from the sites were detected, it is important to acknowledge that this SNP analysis was characterized by low statistical power (N = 405 SNPs) and future work using higher resolution multilocus data^93,94^ might detect population differentiation. Regardless, *C. goreaui* showcased significantly different gene expression patterns based on source location (Fig. 3D), suggesting that algae from these locations are functionally different, even though host gene expression did not exhibit this signal. Functional differences between *C. goreaui* from these same NS and FR locations have previously been reported, whereby algae associated with FR-sourced corals exhibited constitutively higher photochemical efficiency and enrichment of genes associated with photosynthesis^66^. While it is perhaps surprising that *C. goreaui* maintained distinct expression profiles 3.5 years post-transplantation, these same algae also had constitutively different gene expression profiles even after a 95-day thermal and CO_2_-acidification stress experiment^66^. This maintenance of functional differences suggests that *S. siderea* is unable to shuffle its symbiont composition during transplantation, although it is possible that bleaching is necessary for symbiont shuffling. Regardless, this lack of shuffling is interesting given that symbiont shuffling has been suggested as a broad acclimatization strategy for other coral species^95^.

Here we show that algal symbionts associated with BR-sourced corals exhibited underrepresentation of photosynthesis-associated GO terms relative to algae associated with both FR- and NS-sourced corals. Previous work on corals from these same MBRS reefs showcased enrichment of photosynthesis genes in algae associated with corals from the FR relative to the NS (BR corals were not investigated)^66^. This same study also demonstrated that FR corals whose algae were enriched for photosynthesis-related GO terms were more susceptible to thermal challenge with NS *S. siderea* bleaching less than FR corals under heat stress^66^. Together, data from our study coupled with patterns from Davies et al.^65^ may suggest that corals from these environments differ in their thermal tolerance. Differences in thermal tolerance between corals from different environments have also been observed for *P. astreoides* in Florida, although this thermal tolerance was more strongly linked to coral host genetics^20^. While the coral host’s role in thermal tolerance has been shown for many coral species^25,26^, Symbiodiniaceae algae also play an important role^96^. Overall, it is clear that thermal tolerance is a complex trait that is highly dependent on different members of the coral holobiont, which likely depends on coral life history^97^, environment^98^, and symbiont community diversity^96^ and future work conducting thermal challenge experiments after reciprocal transplantation would be interesting.

We also observed a significant effect of transplant location on *C. goreaui* gene expression, although this effect was only detected in algae associated with corals transplanted to the NS. This pattern suggests that algae living in the NS environment require different gene repertoires to function. Indeed, we observed that algal gene expression plasticity was particularly high for algae associated with NS-transplanted corals. These changes in *C. goreaui* gene expression may be driven by lower light levels on NS reefs^78^ because algae associated with BR- and FR-transplanted corals, where light levels are likely higher than NS environments^33,99^, exhibit more similar gene expression. Additionally, these differences in expression observed in NS-transplanted *C. goreaui* may be associated with other environmental factors (e.g., temperature, salinity) acting on the coral holobiont that may impact the animal-algae symbiosis at the molecular level^23,100^. However, it is important to emphasize that the *C. goreaui* gene expression patterns reported here are based on low depth of coverage recovered from Tagseq data. Future work assessing functional differences between coral-associated Symbiodiniaceae should target algal cells during RNA isolation, which would increase algal coverage and result in more robust conclusions.

In conclusion, transplantation to novel reef environments revealed evidence of environmental specialization and/or long-term acclimation in FR-sourced *S. siderea* in southern Belize that was not observed in either NS- or BR-sourced populations. In addition, if the colony survived, we observe that these populations exhibit strong capacities for gene expression plasticity when introduced to novel reef environments. Given that *S. siderea* can live for hundreds of years^101^, it likely evolved the ability to deal with environmental change well before climate and oceanic change intensified due to human activity, so this capacity for plasticity is perhaps not surprising. Further, the high, but incomplete, mortality observed in the NS environment suggests that understanding genetic variation of survival in more variable environments should be a priority to better predict *S. siderea* responses to persistent environmental change. Together, these findings help explain why *S. siderea* remains a dominant reef-building coral on Caribbean reefs, despite the impact of intensifying local and global stressors on these ecosystems. As legislators and coral reef managers continue developing plans for the protection and restoration of Caribbean coral reefs, we urge them to consider the role that the diverse physiological abilities of different populations of *S. siderea*, and similar coral species, could play in sustaining these ecosystems during this interval of rapid environmental change.

### Supplementary Information

Supplementary Information.

## Data Availability

Raw reads for all 42 gene expression samples and 29 ITS2 metabarcoding data are available on the NCBI Short Read Archive (SRA) under BioProject number PRJNA938378. All other scripts and code required to generate gene expression, calcification, survival, and ITS2 results can be found on GitHub at https://github.com/seabove7/BelizeRT_Castillo_Bove and Zenodo (10.5281/zenodo.7739118). Reviewer-only access to SRA projects (will expire on release).
