# Supplementary material for: Gene expression plasticity facilitates acclimatization of a long-lived Caribbean coral across divergent reef environments
Source: Sci Rep. 2024 Apr 3;14:7859. doi: 10.1038/s41598-024-57319-0 (PMC10991280; doi:10.1038/s41598-024-57319-0)
Supplement: Supplementary file 1 — Supplementary Information. [file 41598_2024_57319_MOESM1_ESM.pdf]

# Supplemental Materials

Supporting materials for the manuscript, "*Gene expression plasticity facilitates acclimatization of a long-lived Caribbean coral across divergent reef environments*" by Castillo/Bove et al.

Karl D. Castillo[1,2#*], Colleen B. Bove[2,3#*], Annabel M. Hughes[3], Maya E. Powell[2], Justin B. Ries[4], Sarah W. Davies[2,3*]

[1]Department of Earth, Marine and Environmental Sciences, University of North Carolina at Chapel Hill, Chapel Hill, NC, USA
[2]Environment, Ecology and Energy Program, University of North Carolina at Chapel Hill, Chapel Hill, NC, USA
[3]Department of Biology, Boston University, Boston, MA, USA
[4]Department of Marine and Environmental Sciences, Marine Sciences Center, Northeastern University, Nahant, MA, USA
*# Co-first Authors*
** Corresponding authors*

## Supplemental Figures

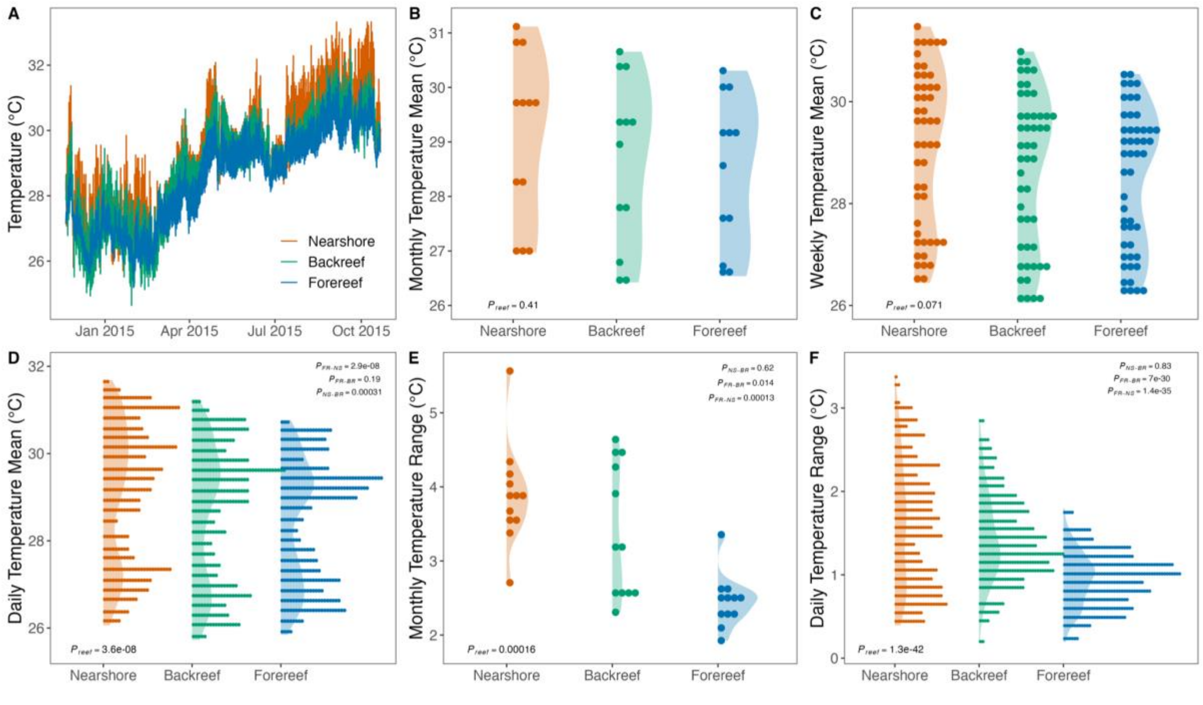


**Figure S1**. *In situ* temperatures recorded every 30 minutes from November 2014 to October 2015 on the nearshore (NS; orange), backreef (BR; green), and forereef (FR; blue) habitats assessed during this reciprocal transplant experiment on the Belize MBRS. (**A**) Raw temperature observations showing overall temperature patterns through the sampling period at each site. (**B**) Monthly, (**C**) Weekly, and (**D**) daily mean temperature comparisons across the three reef environments. (**E**) Monthly and (**F**) daily temperature ranges across the three reef environments demonstrating differences in temperature variability across sites.

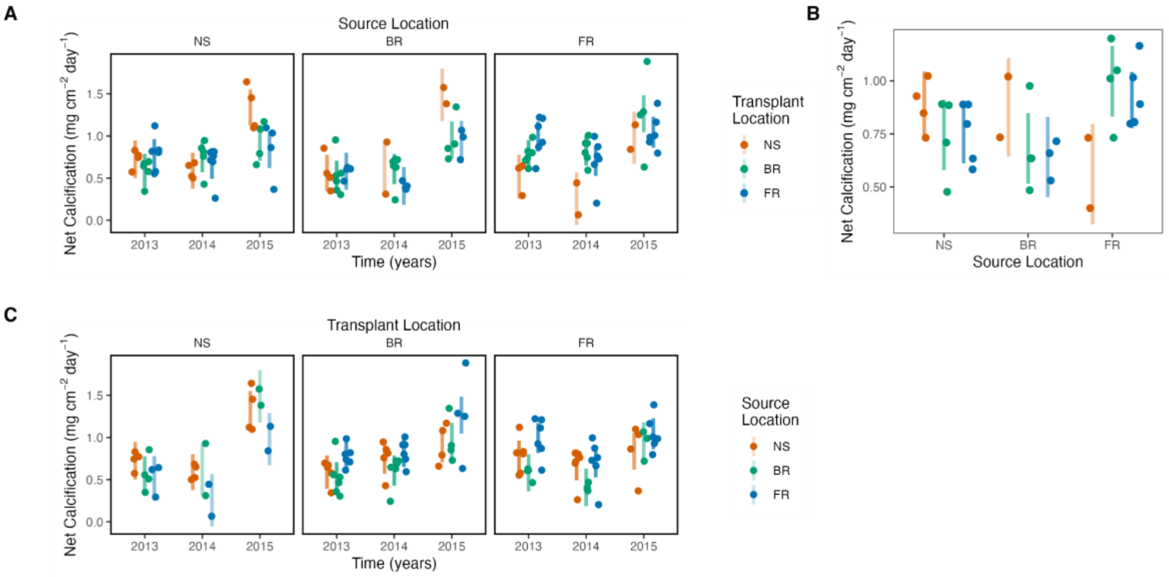


**Figure S2**. Calcification rate (mg cm$^{-2}$ day$^{-1}$) coloured by either (**A-B**) transplant or (**C**) source location (orange = NS [nearshore], green = BR [backreef], and blue = FR [forereef]), at (**A, C**) yearly intervals or (**B**) averaged over the entire transplant experimental period. Colored bars represent modeled 95% confidence intervals with corresponding raw calcification rates per colony denoted by circles of the same color.

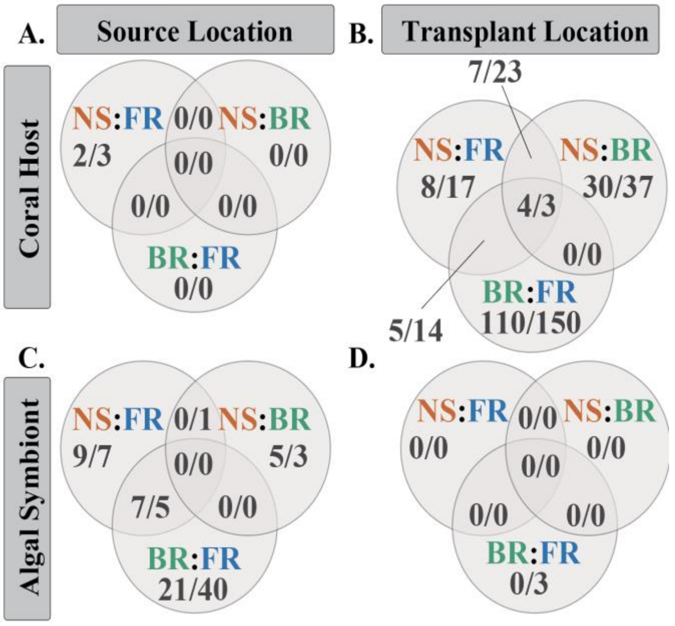


**Figure S3.** Venn diagram of differentially expressed genes (DEGs) unique and shared (intersection) between each pairwise comparison across source location (A, C) and transplant location (B, D) for *Siderastrea siderea* (A, B) and *Cladocopium goreaui* algal symbionts *in hospite* (C, D). The first number is upregulated in the first site of the comparison and the second number is upregulated in the second site listed. NS = nearshore, BR = backreef, and FR = forereef.

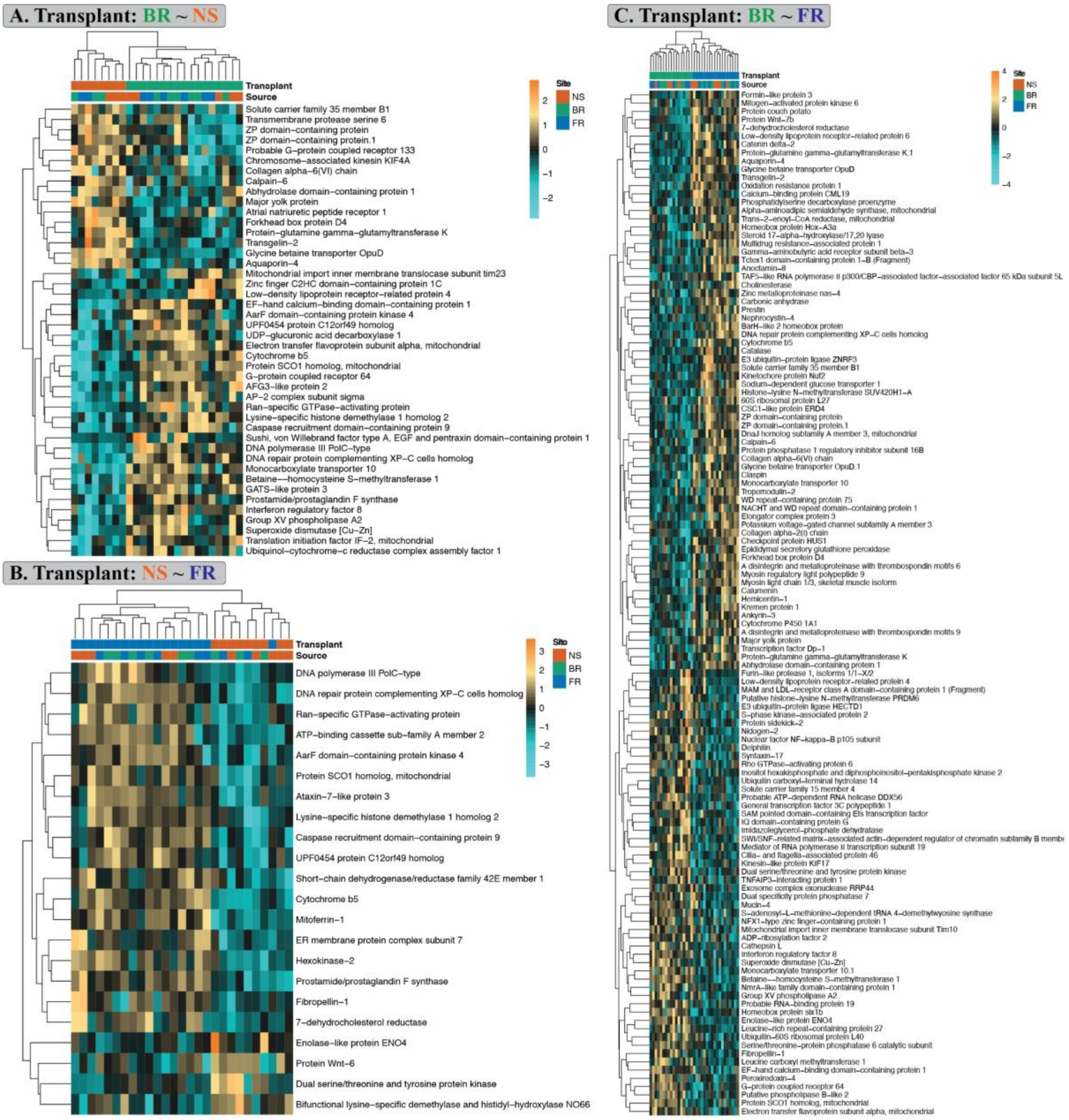


**Figure S4.** Differentially expressed genes (DEGs, FDR adjusted *p*-value < 0.10) with annotation of *Siderastrea siderea* between pairwise transplant locations: A. Backreef (BR) ~ Nearshore (NS), B. NS ~ Forereef (FR), and C. BR ~ FR. Heatmap showing annotated DEGs where each row is a gene and each column is a unique transcriptome library. The color scale is in log2 (fold change relative to the gene's mean) and genes and samples are clustered hierarchically based on Pearson's correlation of their expression across samples. Orange, green and blue blocks indicate both the transplant location (top) and the source location (bottom). Hierarchical clustering of libraries (columns) demonstrates strong differences in gene expression by transplant location.

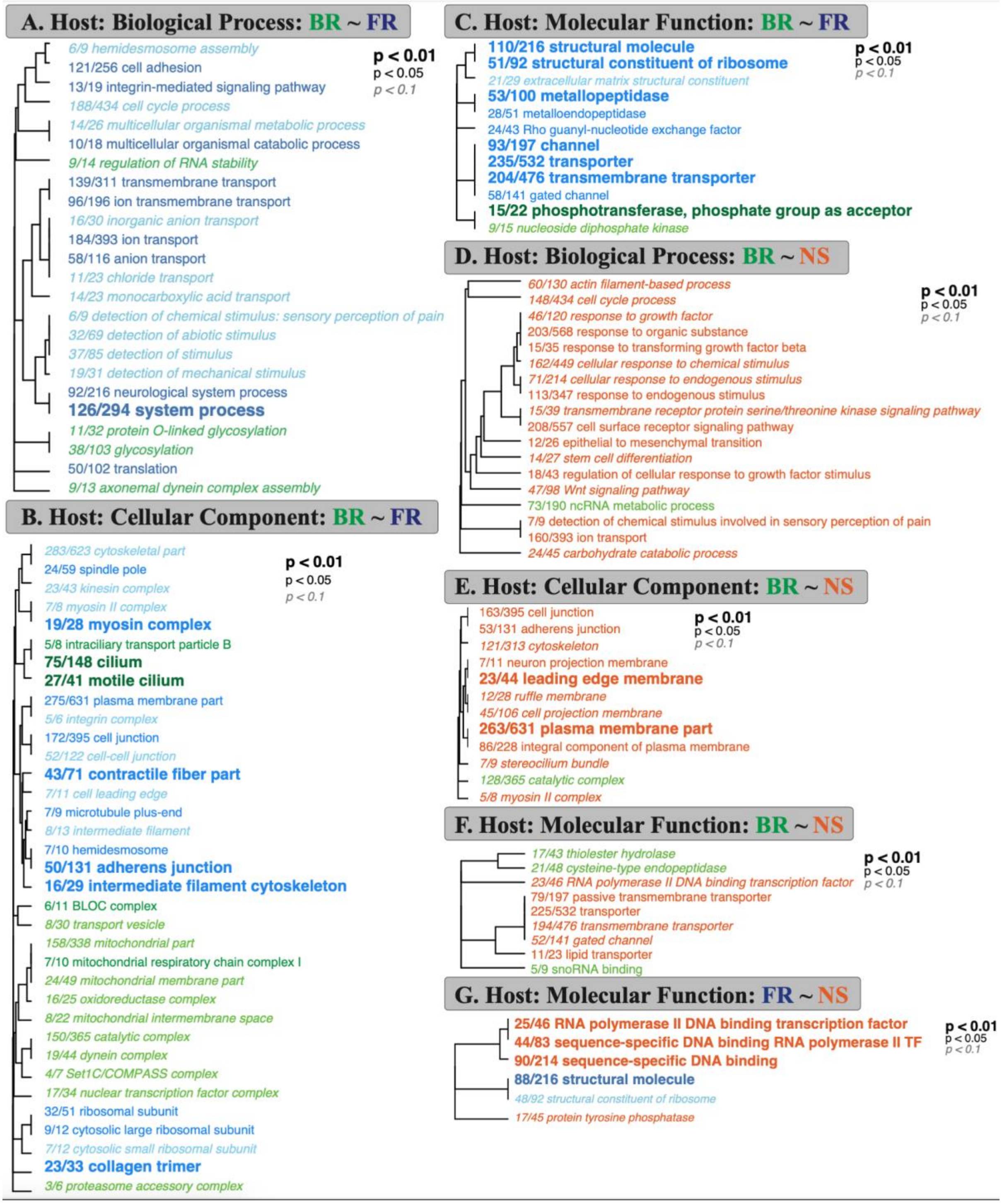


**Figure S5.** GO enrichment of expression patterns of *Siderastrea siderea* between pairwise transplant locations (Nearshore (NS), Backreef (BR), Forereef (FR). Gene ontology (GO) enrichment of "Biological process (BP), "Cellular component (CC)", and Molecular Function (MF) categories derived from the gene expression differences across transplant locations. Dendrograms depict sharing of genes between categories (categories with no branch length between them are subsets of each other), with the fractions corresponding to proportion of genes with an unadjusted $p < 0.05$ relative to the total number of genes within the category. Text size and boldness indicate the significance (Mann–Whitney $U$ tests) of the term. Orange categories are enriched in NS, green enriched in BR, and blue enriched in FR.

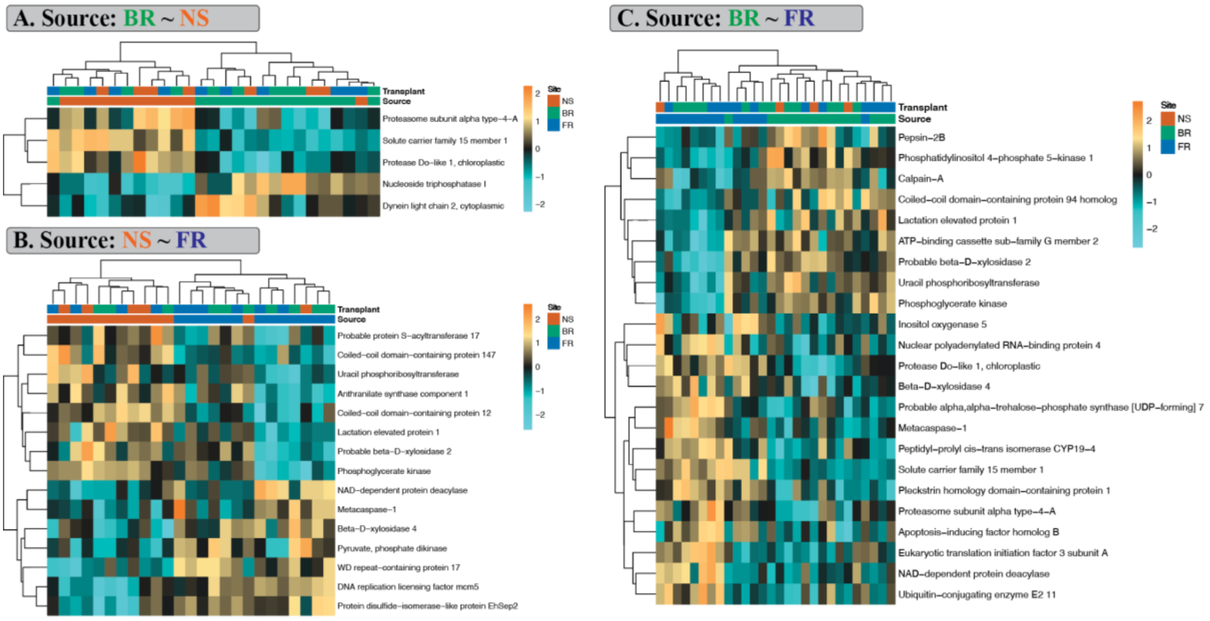


**Figure S6.** Differentially expressed genes (DEGs, FDR adjusted *p*-value < 0.10) with annotation of *Cladocopium goreaui* between pairwise source locations: A. Backreef (BR) ~ Nearshore (NS), B. NS ~ Forereef (FR), and C. BR ~ FR. Heatmap showing annotated DEGs where each row is a gene and each column is a unique transcriptome library. The color scale is in log2 (fold change relative to the gene's mean) and genes and samples are clustered hierarchically based on Pearson's correlation of their expression across samples. Orange, green and blue blocks indicate both the transplant location (top) and the source location (bottom). Hierarchical clustering of libraries (columns) demonstrates strong differences in gene expression by source location.

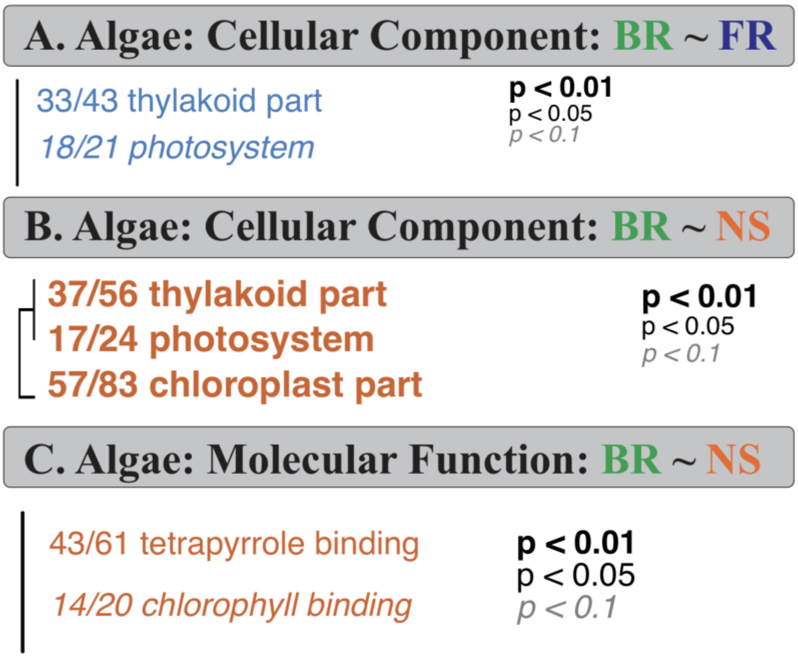


**Figure S7.** GO enrichment of expression patterns of *Cladocopium goreaui* between pairwise source locations (Nearshore (NS), Backreef (BR), Forereef (FR). Gene ontology (GO) enrichment of "Cellular component (CC)" and Molecular Function (MF) categories derived from the gene expression differences across transplant locations. No enriched GO terms were observed for Biological Process (BP). Dendrograms depict sharing of genes between categories (categories with no branch length between them are subsets of each other), with the fractions corresponding to proportion of genes with an unadjusted $p < 0.05$ relative to the total number of genes within the category. Text size and boldness indicate the significance (Mann–Whitney *U* tests) of the term. Orange categories are enriched in NS, green enriched in BR, and blue enriched in FR.

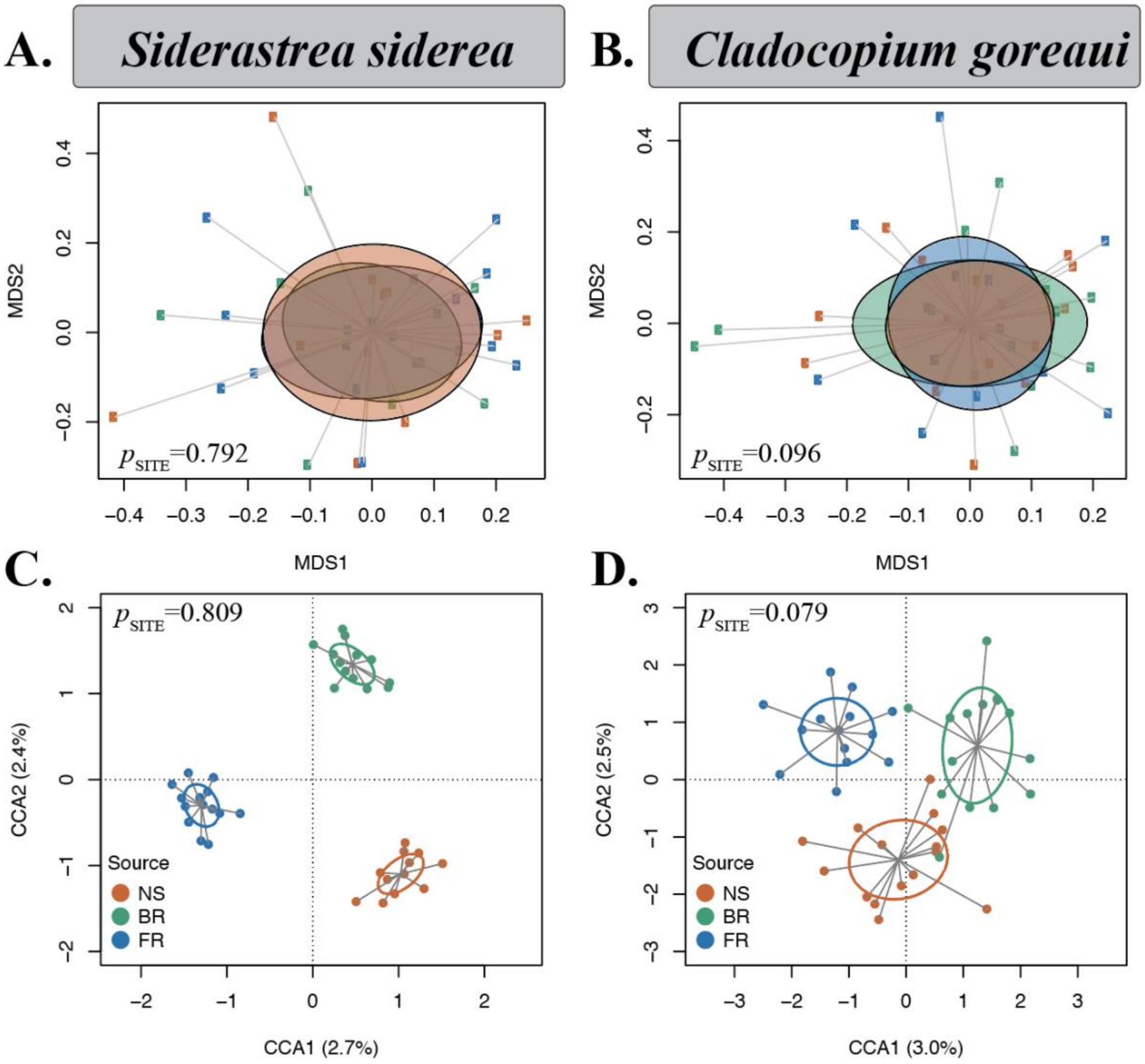


**Figure S8.** Multidimensional scaling (MDS) (A,B) and Canonical correspondence analysis (C,D) plots based on genetic covariance matrices of all samples for *Siderastrea siderea* (A, C) and *Cladocopium goreaui* (B, D), highlighting that there is no genetic structure between hosts or algal symbionts between different source locations (Orange= Nearshore (NS), green = Backreef (BR), blue = Forereef (FR)) regardless of unconstrained or constrained analyses.

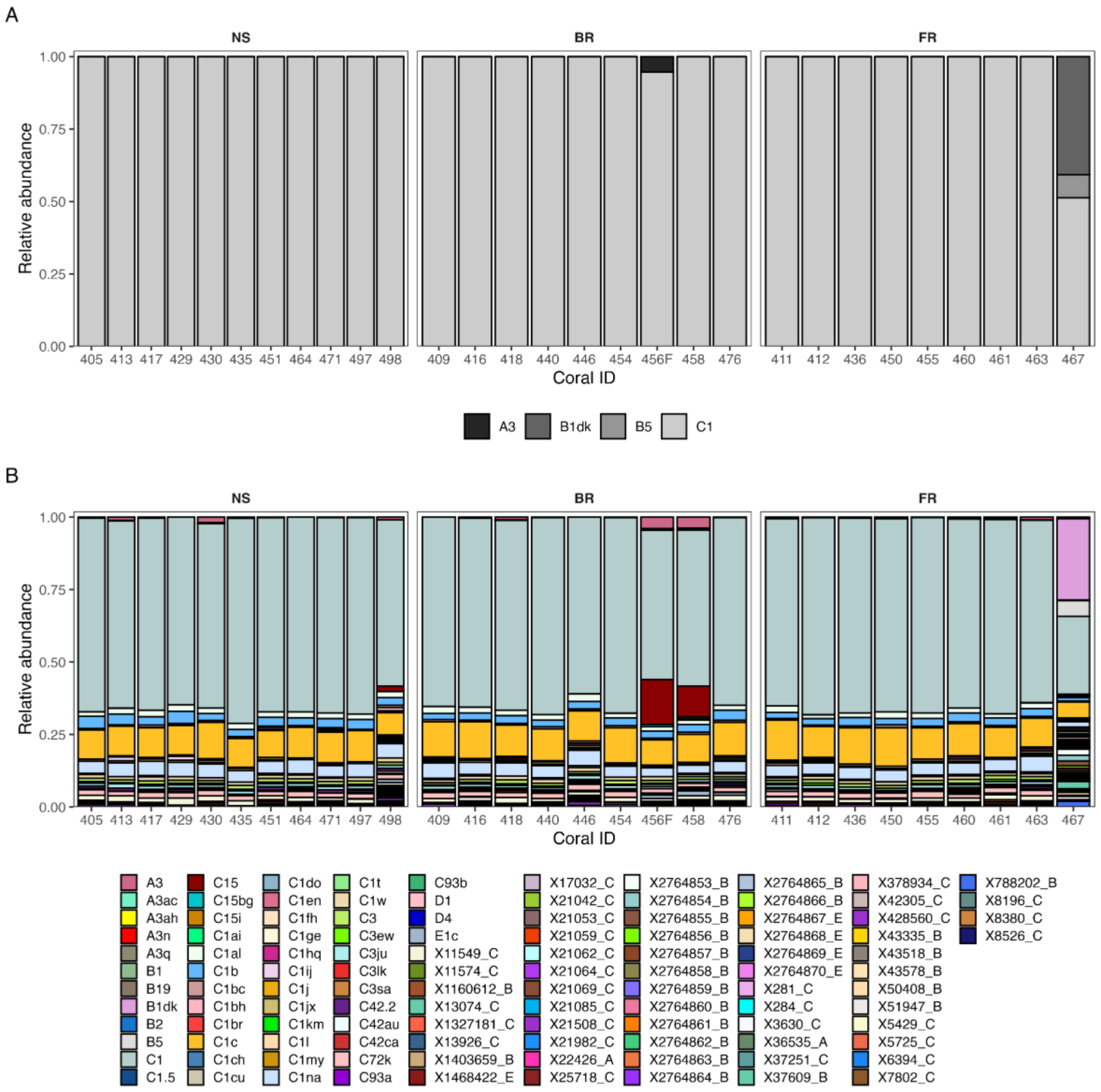


**Figure S9**. Relative abundance of major ITS2 types (A) as well as the post-med sequences (B) within each coral colony grouped by source location. The dominant algal symbiont species was determined to be *Cladocopium goreaui* (C1) for all collected corals.

## Supplemental Tables

**Table S1**. **Akaike information criterion (AIC) model selection for calcification linear models.** Model selection for assessment of source and transplant location impacts on overall (2012 - 2015) and annual coral calcification rates performed using AIC. The model with the lowest AIC (in bold) best fit the data and was used for analyses.

| | **df** | **AIC** |
|---|---|---|
| ***Overall Calcification*** | | |
| **Source * Transplant** | **10** | **-15.05** |
| Source + Transplant | 6 | -8.51 |
| Transplant only | 4 | -7.76 |
| Source only | 4 | -12.34 |
| ***Annual Calcification*** | | |
| **Source * Transplant * Year** | **28** | **6.48** |
| Source + Transplant + Year | 8 | 19.38 |
| Source * Transplant + Year | 12 | 10.25 |
| Source * Year + Transplant | 12 | 24.21 |
| Transplant * Year + Source | 12 | 6.24 |
| Transplant only | 4 | 67.55 |
| Source only | 4 | 61.23 |
| Year only | 4 | 19.68 |

**Table S2**. Summary of gene expression data

| Sample | Source | Transplant | Raw Reads | Trimmed Reads | Duplicate reads | Reads with No Header | Reads with N in header | Host counts | Algal counts |
|---|---|---|---|---|---|---|---|---|---|
| Ssid284 | FR | FR | 8871096 | 2416828 | 6119784 | 334096 | 388 | 519518 | 63016 |
| Ssid401# | NS | FR | 6584522 | 1829474 | 4436686 | 318084 | 278 | 323215 | 38890 |
| Ssid402 | FR | BR | 11117973 | 3280980 | 7410108 | 426373 | 512 | 600920 | 168102 |
| Ssid405 | NS | FR | 12466424 | 2805055 | 9167759 | 493010 | 600 | 550094 | 67775 |
| Ssid409 | BR | FR | 13137844 | 3651068 | 8919528 | 566643 | 605 | 743124 | 79255 |
| Ssid411 | FR | NS | 9745182 | 2892508 | 6419824 | 432426 | 424 | 547549 | 59781 |
| Ssid412 | FR | BR | 9413272 | 2265367 | 6784285 | 363173 | 447 | 397676 | 89074 |
| Ssid413* | NS | FR | 11870502 | 2403145 | 9019805 | 446988 | 564 | 434612 | 68982 |
| Ssid415 | FR | BR | 9609736 | 2664087 | 6508584 | 436630 | 435 | 485601 | 65403 |
| Ssid416 | BR | NS | 8038119 | 2576355 | 5106336 | 355064 | 364 | 523617 | 54596 |
| Ssid417 | NS | NS | 8191125 | 2108608 | 5751937 | 330214 | 366 | 414490 | 49852 |
| Ssid418 | BR | BR | 9826134 | 2864116 | 6563853 | 397750 | 415 | 613307 | 55579 |
| Ssid429 | NS | FR | 11258451 | 2744157 | 8039425 | 474365 | 504 | 496887 | 78184 |
| Ssid430 | NS | FR | 12618488 | 2715266 | 9446399 | 456247 | 576 | 534664 | 79425 |
| Ssid435 | NS | NS | 11103393 | 2772411 | 7896140 | 434365 | 477 | 564075 | 61490 |
| Ssid436 | FR | FR | 8122133 | 2330741 | 5366141 | 424926 | 325 | 430586 | 60565 |
| Ssid438 | BR | BR | 11031208 | 3318588 | 7261402 | 450757 | 461 | 651026 | 96726 |
| Ssid440 | BR | NS | 8533254 | 1923207 | 6285533 | 324103 | 411 | 331336 | 41859 |
| Ssid444 | FR | FR | 10586308 | 2579150 | 7566627 | 440039 | 492 | 503790 | 72573 |
| Ssid445 | BR | FR | 10606688 | 2986764 | 7098838 | 520627 | 459 | 478136 | 93115 |
| Ssid446 | BR | BR | 11674748 | 3026021 | 8231314 | 416925 | 488 | 613227 | 72815 |
| Ssid447# | NS | FR | 6763825 | 1922110 | 4503508 | 337938 | 269 | 321495 | 33569 |
| Ssid450 | FR | BR | 9939515 | 2208911 | 7297402 | 432736 | 466 | 394210 | 48953 |
| Ssid451 | NS | BR | 10505985 | 2771511 | 7286250 | 447756 | 468 | 545799 | 61487 |
| Ssid454 | BR | BR | 9164134 | 2450703 | 6355810 | 357188 | 433 | 506455 | 55569 |
| Ssid455 | FR | FR | 10813813 | 2684227 | 7694301 | 434807 | 478 | 522783 | 61426 |
| Ssid456F | BR | FR | 13316044 | 3366428 | 9386356 | 562662 | 598 | 701497 | 63886 |
| Ssid457 | BR | FR | 12037182 | 3136316 | 8376885 | 523435 | 546 | 604132 | 77722 |
| Ssid458 | BR | NS | 8360531 | 2326960 | 5705076 | 328096 | 399 | 463545 | 52855 |
| Ssid460 | FR | BR | 9877316 | 2749847 | 6679859 | 447167 | 443 | 534075 | 68271 |
| Ssid461 | FR | BR | 10244623 | 2854421 | 6975045 | 414662 | 495 | 555605 | 90576 |
| Ssid463 | FR | FR | 10351636 | 2193733 | 7735862 | 421560 | 481 | 436165 | 54794 |
| Ssid464^ | NS | BR | 9920318 | 2555895 | 6944924 | 419052 | 447 | 492648 | 64890 |
| Ssid467# | FR | NS | 9534951 | 2276864 | 6852597 | 405069 | 421 | 427439 | 29442 |
| Ssid470^ | BR | FR | 11326724 | 2456019 | 8435193 | 435026 | 486 | 485561 | 69572 |
| Ssid471^ | NS | BR | 11107934 | 2722828 | 7887553 | 497043 | 510 | 516245 | 49637 |
| Ssid472^ | BR | BR | 9999096 | 2381226 | 7106149 | 511295 | 426 | 409621 | 106843 |
| Ssid476* | BR | FR | 10150995 | 2027428 | 7717478 | 405590 | 499 | 390309 | 44312 |
| Ssid477 | NS | BR | 9839847 | 2665129 | 6795860 | 378397 | 461 | 497179 | 77713 |
| Ssid495 | FR | FR | 10284061 | 2485694 | 7391499 | 406409 | 459 | 484996 | 91491 |
| Ssid497 | NS | NS | 10379851 | 2506321 | 7402653 | 470390 | 487 | 434186 | 56860 |
| Ssid498# | NS | BR | 6477963 | 1901045 | 4229453 | 347168 | 297 | 301870 | 23375 |
| Ssid499 | BR | BR | 7222832 | 2398167 | 4502818 | 321538 | 309 | 483145 | 50846 |
| SsidUNK | NS | NS | 8825822 | 2082978 | 6345161 | 397282 | 401 | 424235 | 43552 |

* potential clonal pair
^ outliers in host population genetic analysis
# outliers in symbiont GE analysis

**Table S3**. Akaike information criterion (AIC) model selection for coral host and algal symbiont gene expression plasticity. Model selection for assessment of source and transplant location impacts on gene expression plasticity performed using AIC.

| | AIC | BIC | Nagelkerke's $R^2$ |
|---|---|---|---|
| **Host Model** | | | |
| Source * Transplant | 46.845 | 64.687 | 0.833 |
| Source + Transplant | 102.088 | 112.794 | 0.146 |
| Transplant | 99.632 | 106.769 | 0.108 |
| Source | 102.398 | 109.535 | 0.034 |
| **Symbiont Model** | | | |
| Source * Transplant | 31.742 | 46.714 | 0.863 |
| Source + Transplant | 94.949 | 104.93 | 0.038 |
| Transplant | 91.827 | 98.481 | 0.011 |
| Source | 91.317 | 97.971 | 0.027 |

**Table S4**. Number or corals surviving per year in each transplant location (BR = backreef, FR = forereef, NS = Nearshore).

| Source | Transplant | 2011 | 2012 | 2013 | 2014 | 2015 |
|---|---|---|---|---|---|---|
| BR | BR | 6 | 6 | 6 | 6 | 6 |
| BR | FR | 6 | 6 | 6 | 5 | 5 |
| BR | NS | 6 | 4 | 3 | 3 | 3 |
| FR | BR | 6 | 6 | 6 | 6 | 6 |
| FR | FR | 6 | 6 | 6 | 6 | 6 |
| FR | NS | 6 | 3 | 2 | 2 | 2 |
| NS | BR | 6 | 6 | 6 | 6 | 6 |
| NS | FR | 6 | 6 | 6 | 6 | 6 |
| NS | NS | 6 | 5 | 4 | 4 | 4 |

**Table S5**. Statistical assessment of the cox proportional hazards survival model. (**A**) Type 2 analysis of deviance of the overall effect of transplant and source. (**B**) Cox proportional hazard model output, with exp(coef) representing the hazard ratio, se(coef) is the standard error, z is the z score, and Pr(>|z|) is the probability that the estimate could be 0.

| ***(A)*** | **LR Chisq** | **Df** | **Pr(>Chisq)** |
|---|---|---|---|
| Transplant | 19.831 | 2 | 4.94E-05 |
| Source | 1.622 | 2 | 0.4444 |

| ***(B)*** | **Coefficient** | **Exp(coef)** | **SE(coef)** | **z** | **Pr(>\|z\|)** |
|---|---|---|---|---|---|
| Transplant (FR vs BR) | -18.88 | 0.00 | 12566.10 | 0.00 | 1.00 |
| Transplant (FR vs NS) | 2.58 | 13.25 | 1.06 | 2.44 | 0.01 |
| Source (FR vs BR) | -0.17 | 0.85 | 0.71 | -0.23 | 0.81 |
| Source (FR vs NS) | -1.00 | 0.37 | 0.87 | -1.15 | 0.25 |

**Table S6.** Linear model output assessing overall calcification responses. The intercept was set at corals from the backreef transplanted back to the backreef. (BR = backreef, FR = forereef, NS = Nearshore)

| | **Estimate** | **Std. Error** | **t value** | **Pr(>\|t\|)** |
|---|---|---|---|---|
| (Intercept) | 0.68 | 0.08 | 8.1 | 0 |
| source (FR) | 0.32 | 0.12 | 2.65 | 0.01 |
| source (NS) | 0.06 | 0.12 | 0.49 | 0.63 |
| transplant (FR) | -0.05 | 0.13 | -0.37 | 0.72 |
| transplant (NS) | 0.19 | 0.15 | 1.33 | 0.2 |
| source (FR):transplant (FR) | -0.04 | 0.17 | -0.22 | 0.83 |
| source (NS):transplant (FR) | 0.07 | 0.17 | 0.38 | 0.71 |
| source (FR):transplant (NS) | -0.63 | 0.21 | -3.03 | 0.01 |
| source (NS):transplant (NS) | -0.05 | 0.19 | -0.28 | 0.78 |

**Table S7**. Modeled mean calcification rate and 95% confidence interval. Parametric bootstrapped mean and 95% confidence intervals of calcification rates (mg $cm^{-2}$ $day^{-1}$) in response to transplant treatment (BR = backreef, FR = forereef, NS = Nearshore).

| Treatment | Modeled Mean | Lower 95% CI | Upper 95% CI |
|---|---|---|---|
| BR to BR | 0.68 | 0.52 | 0.85 |
| BR to FR | 0.64 | 0.45 | 0.83 |
| BR to NS | 0.88 | 0.64 | 1.11 |
| FR to BR | 1 | 0.83 | 1.16 |
| FR to FR | 0.91 | 0.78 | 1.04 |
| FR to NS | 0.56 | 0.32 | 0.8 |
| NS to BR | 0.74 | 0.58 | 0.91 |
| NS to FR | 0.76 | 0.61 | 0.9 |
| NS to NS | 0.88 | 0.72 | 1.04 |

**Table S8.** Linear model output assessing annual calcification responses. The intercept was set at corals from the backreef (BR) transplanted back to the backreef from 2013.

| | Estimate | Std. Error | t value | Pr(>\|t\|) |
|---|---|---|---|---|
| (Intercept) | 0.53 | 0.09 | 5.79 | 0 |
| source (FR) | 0.25 | 0.13 | 1.94 | 0.06 |
| source (NS) | 0.06 | 0.14 | 0.44 | 0.66 |
| transplant (FR) | 0.05 | 0.14 | 0.34 | 0.74 |
| transplant (NS) | 0.04 | 0.14 | 0.27 | 0.78 |
| Year (2014) | 0.08 | 0.13 | 0.63 | 0.53 |
| Year (2015) | 0.43 | 0.14 | 2.98 | 0 |
| source (FR):transplant (FR) | 0.16 | 0.19 | 0.85 | 0.4 |
| source (NS):transplant (FR) | 0.15 | 0.2 | 0.75 | 0.45 |
| source (FR):transplant (NS) | -0.3 | 0.21 | -1.4 | 0.17 |
| source (NS):transplant (NS) | 0.1 | 0.21 | 0.5 | 0.62 |
| source (FR):Year (2014) | -0.03 | 0.18 | -0.17 | 0.87 |
| source (NS):Year (2014) | 0.09 | 0.19 | 0.48 | 0.63 |
| source (FR):Year (2015) | 0.06 | 0.2 | 0.27 | 0.79 |
| source (NS):Year (2015) | -0.09 | 0.21 | -0.44 | 0.66 |
| transplant (FR):Year (2014) | -0.25 | 0.2 | -1.23 | 0.22 |
| transplant (NS):Year (2014) | -0.03 | 0.23 | -0.13 | 0.9 |
| transplant (FR):Year (2015) | -0.08 | 0.22 | -0.36 | 0.72 |
| transplant (NS):Year (2015) | 0.48 | 0.24 | 1.99 | 0.05 |
| source (FR):transplant (FR):Year (2014) | -0.09 | 0.27 | -0.32 | 0.75 |
| source (NS):transplant (FR):Year (2014) | -0.03 | 0.28 | -0.11 | 0.91 |
| source (FR):transplant (NS):Year (2014) | -0.28 | 0.34 | -0.85 | 0.4 |
| source (NS):transplant (NS):Year (2014) | -0.28 | 0.31 | -0.9 | 0.37 |
| source (FR):transplant (FR):Year (2015) | -0.35 | 0.3 | -1.19 | 0.24 |
| source (NS):transplant (FR):Year (2015) | -0.2 | 0.31 | -0.66 | 0.51 |
| source (FR):transplant (NS):Year (2015) | -0.5 | 0.35 | -1.43 | 0.16 |
| source (NS):transplant (NS):Year (2015) | -0.22 | 0.32 | -0.68 | 0.5 |

**Table S9.** Modeled mean calcification rate and 95% confidence interval by year. Parametric bootstrapped mean and 95% confidence intervals of calcification rates (mg $cm^{-2}$ $day^{-1}$) in response to transplant treatment (BR = backreef, FR = forereef, NS = Nearshore) over time.

| Treatment | Modeled Mean | Lower 95% CI | Upper 95% CI |
|---|---|---|---|
| BR to BR in 2013 | 0.53 | 0.35 | 0.71 |
| BR to BR in 2014 | 0.61 | 0.43 | 0.79 |
| BR to BR in 2015 | 0.96 | 0.74 | 1.17 |
| BR to FR in 2013 | 0.58 | 0.36 | 0.8 |
| BR to FR in 2014 | 0.41 | 0.18 | 0.63 |
| BR to FR in 2015 | 0.93 | 0.67 | 1.18 |
| BR to NS in 2013 | 0.57 | 0.34 | 0.78 |
| BR to NS in 2014 | 0.62 | 0.32 | 0.93 |
| BR to NS in 2015 | 1.48 | 1.18 | 1.8 |
| FR to BR in 2013 | 0.78 | 0.6 | 0.95 |
| FR to BR in 2014 | 0.83 | 0.65 | 1.01 |
| FR to BR in 2015 | 1.26 | 1.05 | 1.48 |
| FR to FR in 2013 | 0.99 | 0.82 | 1.17 |
| FR to FR in 2014 | 0.7 | 0.53 | 0.88 |
| FR to FR in 2015 | 1.05 | 0.86 | 1.23 |
| FR to NS in 2013 | 0.52 | 0.26 | 0.78 |
| FR to NS in 2014 | 0.25 | -0.06 | 0.57 |
| FR to NS in 2015 | 0.99 | 0.67 | 1.29 |
| NS to BR in 2013 | 0.59 | 0.39 | 0.79 |
| NS to BR in 2014 | 0.76 | 0.57 | 0.96 |
| NS to BR in 2015 | 0.92 | 0.7 | 1.13 |
| NS to FR in 2013 | 0.79 | 0.61 | 0.96 |
| NS to FR in 2014 | 0.67 | 0.49 | 0.86 |
| NS to FR in 2015 | 0.84 | 0.62 | 1.05 |
| NS to NS in 2013 | 0.73 | 0.5 | 0.95 |
| NS to NS in 2014 | 0.59 | 0.38 | 0.8 |
| NS to NS in 2015 | 1.33 | 1.12 | 1.55 |

**Table S10.** Linear model output assessing gene expression plasticity of (A) the coral host and (B) their algal symbionts. The intercept was set at corals from the backreef transplanted back to the backreef. (BR = backreef, FR = forereef, NS = Nearshore)

| | Estimate | Std. Error | t value | Pr(>\|t\|) |
|---|---|---|---|---|
| **A) *Siderastrea siderea* (host)** | | | | |
| *(Intercept)* | -0.67 | 0.17 | -3.87 | 4.6E-04 |
| source (FR) | 1.15 | 0.24 | 4.68 | 4.2E-05 |
| source (NS) | 0.64 | 0.26 | 2.48 | 0.02 |
| transplant (FR) | 1.15 | 0.24 | 4.7 | 4.0E-05 |
| transplant (NS) | 0.08 | 0.3 | 0.26 | 0.80 |
| source (FR):transplant (FR) | -3.04 | 0.35 | -8.79 | 2.2E-10 |
| source (NS):transplant (FR) | -0.26 | 0.35 | -0.72 | 0.47 |
| source (FR):transplant (NS) | 0.12 | 0.46 | 0.26 | 0.80 |
| source (NS):transplant (NS) | -1.55 | 0.41 | -3.75 | 6.5E-04 |
| **B) *Cladocopium spp.* (symbiont)** | | | | |
| *(Intercept)* | -1.26 | 0.14 | -9.29 | 1.8E-10 |
| source (FR) | 1.78 | 0.19 | 9.27 | 1.9E-10 |
| source (NS) | 1.58 | 0.21 | 7.37 | 2.7E-08 |
| transplant (FR) | 1.57 | 0.19 | 8.18 | 3.1E-09 |
| transplant (NS) | 2.1 | 0.23 | 8.94 | 4.3E-10 |
| source (FR):transplant (FR) | -2.93 | 0.27 | -10.82 | 4.7E-12 |
| source (NS):transplant (FR) | -1.09 | 0.3 | -3.59 | 1.1E-03 |
| source (FR):transplant (NS) | *NA* | *NA* | *NA* | *NA* |
| source (NS):transplant (NS) | -3.08 | 0.33 | -9.27 | 1.9E-10 |
